# An Advanced Orthotopic Ovarian Cancer Model in Mice for Therapeutic Trials

**DOI:** 10.1155/2016/2585787

**Published:** 2016-03-23

**Authors:** Ying Zhang, Li Luo, Xueling Zheng, Tinghe Yu

**Affiliations:** Key Medical Laboratory of Obstetrics and Gynecology, The Second Affiliated Hospital, Chongqing Medical University, Chongqing 400010, China

## Abstract

A nude mouse received subcutaneous injection of human ovarian cancer cells HO-8910PM to form a tumor, and then the tumor fragment was surgically transplanted to the ovary of a recipient mouse to establish an orthotopic cancer model. Tumors occurred in 100% of animals. A mouse displayed an ovarian mass, ascites, intraperitoneal spread, and lung metastasis at natural death. The mean survival time was 34.1 ± 17.2 days, with median survival time of 28.5 days. The findings indicated that the present mouse model can reflect the biological behavior of advanced human ovarian cancers. This in vivo model can be used to explore therapeutic means against chemoresistance and metastasis, and an effective treatment would prolong the survival time.

## 1. Introduction

Most ovarian cancers are diagnosed at a later stage. The present treatments produce only mild benefits, leading to poorer prognosis. New therapeutic strategies should be developed to improve the clinical outcome, where an animal model that can simulate the biologic property of an advanced human ovarian cancer (i.e., ovarian mass, ascites, intraperitoneal spread, and distant metastasis) plays a critical role.

Subcutaneous transplantation and intraperitoneal injection of cancer cells are yet the most widely adopted in vivo model for ovarian cancer, which can only reflect partial property of human cancers (e.g., the tumor does not occur in the naturally anatomic position, and the formation of primary tumor and metastasis is not in due sequence). An orthotopic model can recapitulate the initiation, formation, and development of human cancers, thereby being a better model. Several modalities have been introduced and limitations include the following: (i) injecting cell suspensions into the ovarian bursa may lead to a leakage; (ii) the tumor take rate varies dramatically; and (iii) spread and/or metastasis do not occur in some models or are with a low incidence [1–3]. These have been decreasing the clinical relevancy of an animal model and limiting its applications.

Here we report an orthotopic model in nude mice. The model displayed an ovarian mass, ascites, intraperitoneal spread, and distant metastasis and can be used to explore therapeutic strategies against advanced ovarian cancers.

## 2. Materials and Methods

### 2.1. Cells

The highly metastatic human ovarian cancer subline HO-8910PM (Cell Bank of Chinese Academy of Sciences, Shanghai, China) was cultured in RPMI 1640 medium (Hyclone, Beijing, China) supplemented with 10% fetal bovine serum (Hyclone), at 37°C and 5% CO_2_ [4]. Cells were trypsinized, washed with phosphate-buffered saline (PBS), centrifuged, and resuspended in PBS. The concentration was adjusted to 1.0 × 10^7^ cells/mL.

### 2.2. Formation of a Subcutaneous Tumor in Nude Mice

The use of animals was approved by Chongqing Medical University (Chongqing, China) in compliance with the Guide for the Care and Use of Laboratory Animals.

Cell suspension (0.2 mL) was injected subcutaneously in the scapular region of a female nude mouse (4–6 weeks; Center of Laboratory Animals, Chongqing Medical University). A 1.0 cm diameter mass formed after about 6 weeks. The tumor was removed, rinsed with cold PBS, and minced into fragments (1 × 1 × 1 mm^3^) for transplantation.

### 2.3. Orthotopic Transplantation

**Figure 1:**
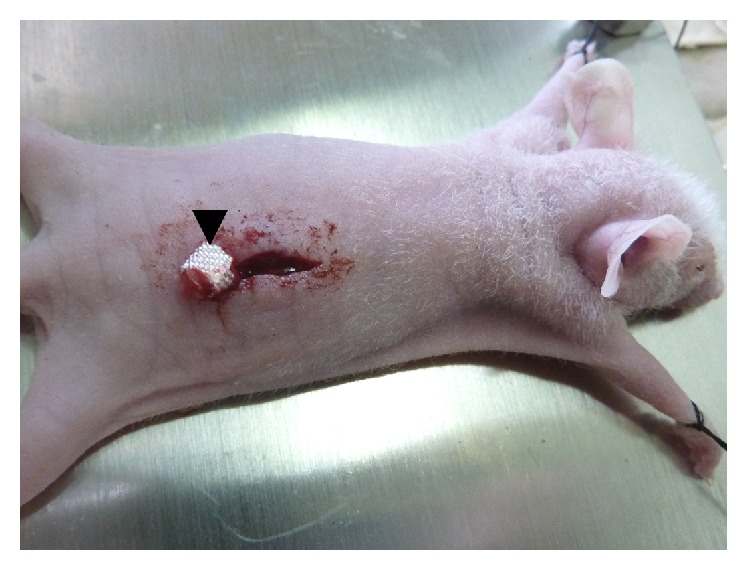
Illustration of the establishment of orthotopic cancer model: the ovary was wrapped with trimmed hemostatic gauze after implanting the cancer fragment.

Orthotopic transplantation was performed in 15 female nude mice. Mice were anesthetized with pentobarbital, and implantation was carried out under a SMZ1000 stereomicroscope (Nikon, Tokyo, Japan). A 1.5 cm dorsal incision was performed in the kidney region. The ovary was partially exteriorized, a slit (1-2 mm) was made, and a cancer graft was introduced into the ovary. The ovary was directly enwrapped with trimmed absorbable hemostatic gauze (the major ingredient was carboxymethyl cellulose; Beijing Tech-Bio-Med Group, Beijing, China) and then replaced into the body cavity (Figure 1). The incision was closed. The tumor formation was examined by abdominal palpation.

Three mice were sacrificed for pathological examinations, and other animals were followed till natural death. The length (*L*), width (*W*), and depth (*D*) of an ovarian tumor were calibrated, and the tumor volume (*V*) was calculated [*V* = (*L* × *W* × *D*)×(*π*/6)].

## 3. Results and Discussion

### 3.1. Tumor Formation

**Figure 2:**
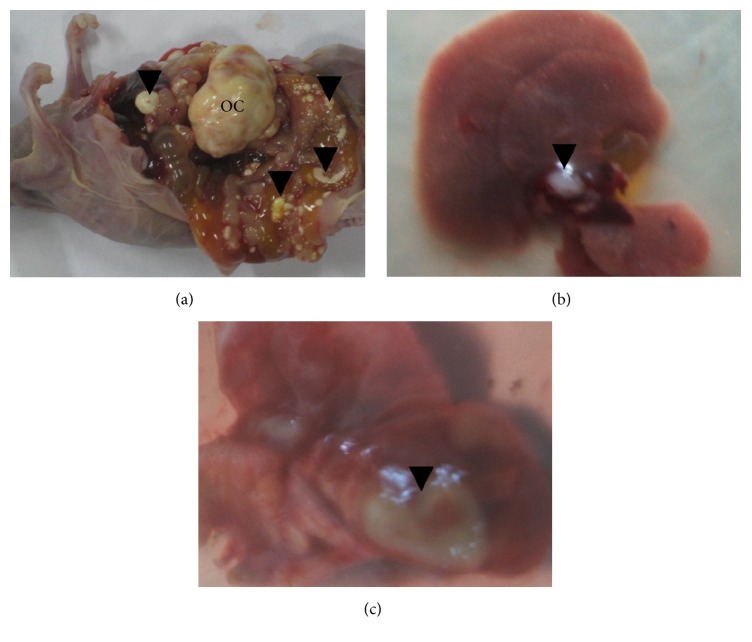
Illustration of ovarian cancer and intraperitoneal spread (a) and of hepatic (b) and pulmonary (c) metastasis. Arrows indicated the metastatic lesions. OC: ovarian cancer.

An abdominal mass (>0.5 cm diameter) can be palpated about 60 days after transplantation. The day was set as day 0 for calculating the survival time. Autopsy indicated that ovarian cancer occurred in all animals; that is, the incidence was 100% (15/15) (Figure 2). The volume of ovarian tumor was 7.2 ± 3.7 cm^3^ (*n* = 12) at natural death.

**Figure 3:**
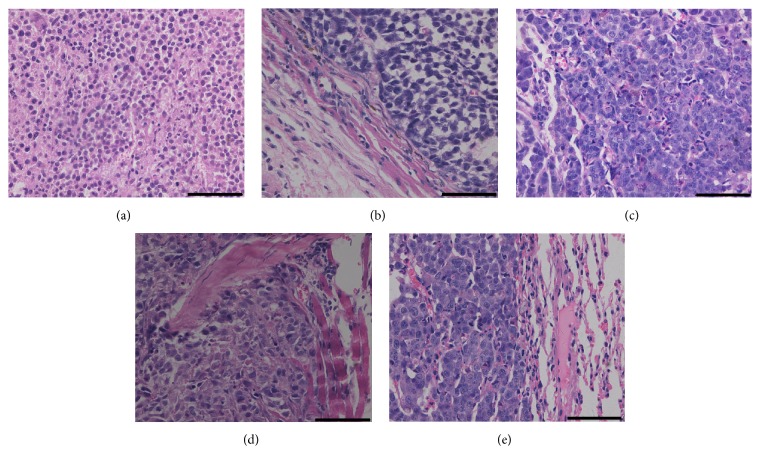
Representative pathological results of ovarian cancer and metastatic foci: ovarian cancer (a) and intraperitoneal (b), hepatic (c), diaphragmatic (d), and pulmonary metastatic lesions (e). The scale was 40 *μ*m.

Autopsy showed that intraperitoneal spread and hemorrhagic ascites occurred in all mice. Metastatic foci were detected in the omentum, intestine, liver, diaphragm, and so forth. Lung metastasis was noted at natural death (Figures 2 and 3). These events frequently occurred in advanced human ovarian cancers, suggesting that the present model can reflect the biologic behavior of a late-stage cancer.

Cancer tissues were usually attached to the ovary with sutures in the conventional protocol, and the detachment of graft fragments led to failure of implantation [1]. The ovary of a mouse was very tiny and fragile. The suture may dilacerate the ovary, resulting in exfoliation of the graft. Indeed, the rate of tumor formation was only 57% in our pilot trials, where the identical cell line was used and the ovary was sutured after introducing the cancer slice. The ovary was directly wrapped with hemostatic gauze in the present study, which provided a physical barrier to protect against displacement of the tumor graft. Cellulose facilitated the formation of clots, in which fibrin and tissue factors promoted cancer angiogenesis [5, 6]. Complete absorption and dissolution of cellulose and the dorsal approach decreased adhesion in the peritoneal cavity. These favored growth of transplanted cancer tissues, thereby increasing the rate of tumor formation.

The subline HO-8910PM owned a high potential of metastasis [4]. Orthotopic implantation provided appropriate microenvironment, favoring growth, invasion, and metastasis of cancer cells [1]. Thus, cancer cells can display their properties thoroughly, resulting in intraperitoneal spread and distant metastasis.

### 3.2. A Model for Experimental Therapy

This model can be used for therapeutic trials (e.g., drug discovery). Additionally, the model can be employed to verify physically focal therapies such as focused ultrasound and electric pulses because there was a relatively larger tumor in the ovary. The considerable variance of tumor volume between mice demonstrated heterogeneity, a phenotype of human ovarian cancer [2]. Thus, the tumor size was not an ideal index to assess the therapeutic efficacy, and the survival time was employed to evaluate the therapeutic response in our investigations [7]. This manner was reasonable considering that prolongation of the survival time was the primary therapeutic endpoint for advanced cancers. Further, a strategy that can reduce the tumor size in an ectopic model may not prolong the survival time in an orthotopic model [8]. Therefore, an orthotopic model should be the first choice considering better predictiveness [3].

A chemoresistant cell line was usually subcloned by exposing parent cells to anticancer drugs with elevating concentrations (i.e., acquired resistance). The resistance would gradually decay without drug pressure and ultimately lost this capacity [9]. This limited establishment of an in vivo chemoresistant model since the formation of a tumor needed at least several weeks. In vivo cancers utilizing those cell lines actually may not be resistant tumors, leading to false positive in exploration of chemosensitizers [8]. HO-8910PM cells owned intrinsic resistance, which was an advantage [10]. The property of intrinsic chemoresistance indicated that the cells' resistance can be preserved in vivo. Intraperitoneal and distant metastases were detected in this model, and the formation of primary tumor was prior to that of metastatic foci (i.e., in due sequence). Therefore, the present model can be used to investigate strategies against chemoresistance and metastasis and to explore the behavior of metastatic foci after treating the primary ovarian lesion.

The survival data was available in 12 mice. The mean survival time was 34.1 ± 17.2 (15–67) days, with median survival time of 28.5 days. When there were 7 groups with 5 mice in each group, prolongation of the mean survival time of 29.2 days reached statistical significance [7]. This can be a reference for setting the observation endpoint in following experiments.

### 3.3. Limitations

Not all cell lines can form orthotopic tumors [3]. Only a cell line was validated in this study, which was a limitation. The cell line adopted should be specifically selected according to the research objective. Recent advance in the orthotopic model was the use of tumor fragments from individual patients, thereby being for personalized medicine [3, 11]. The present protocol may be used to improve the rate of tumor take under those circumstances, which should be investigated in following trials.

## 4. Conclusion

Here an orthotopic ovarian cancer model in nude mice was established. This in vivo model can simulate the biological behavior of an advanced human cancer and can be used to explore therapeutic strategies.
